# Complete genome sequence of *Coraliomargarita akajimensis* type strain (04OKA010-24^T^)

**DOI:** 10.4056/sigs.952166

**Published:** 2010-06-15

**Authors:** Konstantinos Mavromatis, Birte Abt, Evelyne Brambilla, Alla Lapidus, Alex Copeland, Shweta Deshpande, Matt Nolan, Susan Lucas, Hope Tice, Jan-Fang Cheng, Cliff Han, John C. Detter, Tanja Woyke, Lynne Goodwin, Sam Pitluck, Brittany Held, Thomas Brettin, Roxanne Tapia, Natalia Ivanova, Natalia Mikhailova, Amrita Pati, Konstantinos Liolios, Amy Chen, Krishna Palaniappan, Miriam Land, Loren Hauser, Yun-Juan Chang, Cynthia D. Jeffries, Manfred Rohde, Markus Göker, James Bristow, Jonathan A. Eisen, Victor Markowitz, Philip Hugenholtz, Hans-Peter Klenk, Nikos C. Kyrpides

**Affiliations:** 1 DOE Joint Genome Institute, Walnut Creek, California, USA; 2 DSMZ - German Collection of Microorganisms and Cell Cultures GmbH, Braunschweig, Germany; 3 Los Alamos National Laboratory, Bioscience Division, Los Alamos, New Mexico USA; 4 Biological Data Management and Technology Center, Lawrence Berkeley National Laboratory, Berkeley, California, USA; 5 Lawrence Livermore National Laboratory, Livermore, California, USA; 6 HZI – Helmholtz Centre for Infection Research, Braunschweig, Germany; 7 University of California Davis Genome Center, Davis, California, USA

**Keywords:** sphere-shaped, non-motile, non-spore-forming, aerobic, mesophile, Gram-negative, *Puniceicoccaceae*, *Opitutae*, GEBA

## Abstract

*Coraliomargarita akajimensis* Yoon *et al.* 2007 is the type species of the genus *Coraliomargarita*. *C. akajimensis* is an obligately aerobic, Gram-negative, non-spore-forming, non-motile, spherical bacterium that was isolated from seawater surrounding the hard coral *Galaxea fascicularis. C. akajimensis* is of special interest because of its phylogenetic position in a genomically under-studied area of the bacterial diversity. Here we describe the features of this organism, together with the complete genome sequence, and annotation. This is the first complete genome sequence of a member of the family *Puniceicoccaceae*. The 3,750,771 bp long genome with its 3,137 protein-coding and 55 RNA genes is a part of the *** G****enomic* *** E****ncyclopedia of* *** B****acteria and* *** A****rchaea * project.

## Introduction

Strain 04OKA010-24^T^ (DSM 45221 = JCM 23193 = KCTC 12865) is the type strain of the species *Coraliomargarita akajimensis* and was first described in 2007 by Yoon *et al.* [1]. Strain 04OKA010-24^T^ was isolated from seawater surrounding the hard coral *Galaxea fascicularis* L., collected at Majanohama, Akajima, Okinawa, Japan. Yoon *et al.* considered strain *C. akajimensis* 04OKA010-24^T^ to represent a novel species in a new genus belonging to subdivision 4 of the phylum *Verrucomicrobia*. Based on 16S rRNA the phylum *Verrucomicrobia* has been divided into five subdivisions [2]. In the second edition of *Bergey's Manual of Systematic Bacteriology* three subdivisions were included at the rank of family: ‘*Verrucomicrobiaceae*’ (subdivision 1), ‘*Xiphinematobacteriaceae*’ (subdivision 2) and ‘*Opitutaceae*’ (subdivision 4) [3]. There were three identified species in subdivision 4, *Opitutus terrae* [4-6] isolated from soil and the marine bacteria ‘*Fucophilus fucoidanolyticus*’ [7], isolated from a sea cucumber and *Alterococcus agarolyticus* [8], isolated from a hot spring that was originally misclassified as a member of the *Gammaproteobacteria*.

In 2007, coincident to the description of *C. akajimensis*, the class *Opitutae*, which comprises two orders: the order (*Puniceicoccales* containing the family *Puniceicoccaceae* and the order *Opitutales* containing the family *Opitutaceae*) was proposed for the classification of species belonging to subdivision 4 of the phylum ‘*Verrucomicrobia*’ [9]. Besides the genus *Coraliomargarita* [1] the genera *Cerasicoccus* [10], *Pelagicoccus* [11], *Puniceicoccus* [9] belong into the family *Puniceicoccaceae*. Here we present a summary classification and a set of features for *C. akajimensis* 04OKA010-24^T^, together with the description of the complete genomic sequencing and annotation.

## Classification and features

Within the class *Opitutae*, strain *C. akajimensis* 04OKA010-24^T^ shares the highest degree of 16S rRNA gene sequence similarity with *Puniceicoccus vermicola* (88.3%), isolated from the digestive tract of a marine clamworm [5], and *Pelagicoccus croceus* (87.6%) [12], whereas the other members of the class share 84.1 to 87.2% sequence similarity [13]. ‘*Lentimonas marisflavi*’ and ‘*Fucophilus fucoidanolyticus*’ are the closest related cultivable strains (94.0% sequence similarity), whose names are not yet validly published. ‘*Fucophilus fucoidanolyticus’* was isolated from sea cucumbers (*Sticopus japonicus*) and is able to degrade fucoin [14]. GenBank contains also a large number of 16S rRNA sequences with reasonably high sequence similarity from phylotypes (uncultured bacteria) reflecting the problem of efficient culturing of bacteria from the class *Opitutae.* However, only few sequences from genomic and marine metagenomic surveys surpass 90% sequence similarity, indicating that members of the genus *Coraliomargarita* are not widely distributed globally in the habitats screened thus far (status April 2010).

**Figure 1:**
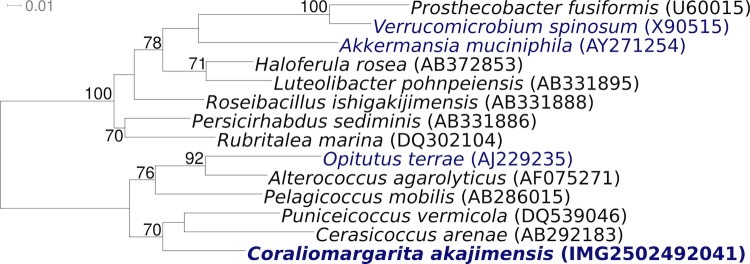
Phylogenetic tree highlighting the position of *C. akajimensis* 04OKA010-24^T^ relative to the other type strains within the phylum *Verrucomicrobia*. The tree was inferred from 1,373 aligned characters [15,16] of the 16S rRNA gene sequence under the maximum likelihood criterion [17] and rooted in accordance with the current taxonomy [18]. The branches are scaled in terms of the expected number of substitutions per site. Numbers above branches are support values from 300 bootstrap replicates [19] if larger than 60%. Lineages with type strain genome sequencing projects registered in GOLD [20] are shown in blue (*Akkermansia muciniphila* CP001071, *Opitutus terrae* CP001032), published genomes in bold.

Figure 1 shows the phylogenetic neighborhood of *C. akajimensis* 04OKA010-24^T^ in a 16S rRNA based tree. The two copies of the 16S rRNA gene in the genome are identical with the previously published sequence generated from DSM 45221 (AB266750).

**Figure 2:**
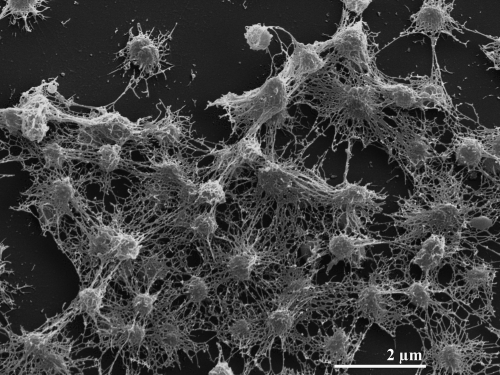
Scanning electron micrograph of *C. akajimensis* 04OKA010-24^T^

Cells of *C. akajimensis* 04OKA010-24^T^ are Gram-negative, obligately aerobic cocci with a diameter of 0.5-1.2 µm (Figure 2 and Table 1) [1]. The cells are non-motile and spores are not formed. On half strength R2A agar medium with 75% artificial seawater *C. akajimensis* forms circular, convex, white colonies. The optimum temperature for growth ranges from 20 to 30°C. No growth was observed at 4 or 45°C. The pH range for growth is 7.0-9.0. NaCl concentrations up to 5% (w/v) are tolerated [1].

**Table 1:** Classification and general features of *C. akajimensis* 04OKA010-24^T^ according to the MIGS recommendations [21].

**MIGS ID**	**Property**	**Term**	**Evidence code**
	Current classification	Domain *Bacteria*	TAS [22]
		Phylum *Verrucomicrobia*	TAS [23,24]
		Class *Opitutae*	TAS [19, 9]
		Order *Puniceicoccales*	TAS [19, 9]
		Family *Puniceicoccaceae*	TAS [19, 9]
		Genus *Coraliomargarita*	TAS [1]
		Species *Coraliomargarita akajimensis*	TAS [1]
		Type strain 04OKA010-24	
	Gram stain	negative	TAS [1]
	Cell shape	sphere-shaped cocci	TAS [1]
	Motility	non-motile	TAS [1]
	Sporulation	non-sporulating	TAS [1]
	Temperature range	mesophile	TAS [1]
	Optimum temperature	20-30°C	TAS [1]
	Salinity	up to 5% NaCl	TAS [1]
MIGS-22	Oxygen requirement	aerobic	TAS [1]
	Carbon source	acid production from mannitol, mannose, galactose, fructose	TAS [1]
	Energy source	chemoorganotrophic	TAS [1]
MIGS-6	Habitat	marine, seawater surrounding the hard coral *Galaxea fascicularis*	TAS [1]
MIGS-15	Biotic relationship	free living	NAS
MIGS-14	Pathogenicity	non pathogenic	NAS
	Biosafety level	1	TAS [25]
	Isolation	seawater	TAS [1]
MIGS-4	Geographic location	Majanohama, Akajima, Okinawa, Japan	TAS [1]
MIGS-5	Sample collection time	March 2004	TAS [1]
MIGS-4.1 MIGS-4.2	Latitude Longitude	39.538 141.122	NAS
MIGS-4.3	Depth	not reported	
MIGS-4.4	Altitude	not reported	

Evidence codes - IDA: Inferred from Direct Assay (first time in publication); TAS: Traceable Author Statement (i.e., a direct report exists in the literature); NAS: Non-traceable Author Statement (i.e., not directly observed for the living, isolated sample, but based on a generally accepted property for the species, or anecdotal evidence). These evidence codes are from the Gene Ontology project [26]. If the evidence code is IDA, then the property was directly observed by one of the authors or an expert mentioned in the acknowledgements.

Strain 04OKA010-24^T^ produces acid from glycerol, galactose, fructose, mannose, mannitol, sorbitol, trehalose, D-turanose, D-lyxose, D-tagatose, D-fucose, L-fucose, D-arabitol, and 5-ketogluconate [1]. *C. akajimensis* is able to hydrolyze urea and DNA, but cannot hydrolyze agar, casein, aesculin, starch and gelatin [1]. Nitrate is not reduced to nitrite. *C. akajimensis* is catalase negative, oxidase positive [1] and is resistant to ampicillin and penicillin G [10].

### Chemotaxonomy

The fatty acid profile of strain *C. akajimensis* 04OKA010-24^T^ revealed straight chain acids C_14:0_ (24.2%), C_18:1_ω9c (23.5%) and C_18:0_ (15.6%) as the major fatty acids and iso-C_14:0_ (8.2%), anteiso-C_15:0_ (2.9%), C_16:0_ (3.3%) C_19:0_ (2.8%) and C_21:0_ (6.9%) in minor amounts [1]. MK-7 is the predominant menaquinone [1]. Muramic acid and diaminopimelic acid are absent, indicating that the cell wall does not contain peptidoglycan [1].

## Genome sequencing and annotation

### Genome project history

This organism was selected for sequencing on the basis of its phylogenetic position [27], and is part of the *** G****enomic* *** E****ncyclopedia of* *** B****acteria and* *** A****rchaea * project [28]. The genome project is deposited in the Genome OnLine Database [20] and the complete genome sequence is deposited in GenBank. Sequencing, finishing and annotation were performed by the DOE Joint Genome Institute (JGI). A summary of the project information is shown in Table 2.

**Table 2:** Genome sequencing project information

**MIGS ID**	**Property**	**Term**
MIGS-31	Finishing quality	Finished
MIGS-28	Libraries used	Three genomic libraries: 454 pyrosequence standard library, 454 pyrosequence 24kb PE library and Illumina stdandard library
MIGS-29	Sequencing platforms	454 GS FLX, Illumina GAii
MIGS-31.2	Sequencing coverage	43.5× pyrosequence, 190.3× Illumina
MIGS-30	Assemblers	Newbler version 2.0.0-PostRelease-11/04/2008, phrap
MIGS-32	Gene calling method	Prodigal 1.4, GenePRIMP
	INSDC ID	CP001998
	Genbank Date of Release	April 5, 2010
	GOLD ID	Gc01256
	NCBI project ID	33365
	Database: IMG-GEBA	2502422317
MIGS-13	Source material identifier	DSM 45221
	Project relevance	Tree of Life, GEBA

### Growth conditions and DNA isolation

*C. akajimensis* 04OKA010-24^T^, DSM 45221, was grown in DSMZ medium 514 (bacto marine growth medium) [29] at 25°C. DNA was isolated from 0.5-1 g of cell paste using a MasterPure Gram Positive DNA purification kit (Epicentre MGP04100), adding 5 µl mutanolysin to the standard lysis solution for 40 min at 37°C and a final 35 min incubation on ice after the MPC-step.

### Genome sequencing and assembly

The genome of *C. akajimensis* was sequenced using a combination of Illumina and 454 technologies. An Illumina GAii shotgun library with reads of 714 Mb, a 454 Titanium draft library with average read length of 282 +/- 187.7 bases, and a paired end 454 library with average insert size of 24.632 +/- 6.158 kb were generated for this genome. All general aspects of library construction and sequencing can be found at http://www.jgi.doe.gov/. Draft assembly was based on 3.8 Mb 454 standard and 454 paired end data (498,215 reads). Newbler (Roch, version 2.0.0-PostRelease-10/28/2008) parameters are -consed -a 50 -l 350 -g -m -ml 20. The initial Newbler assembly was converted into a phrap assembly by making fake reads from the consensus and collecting the read pairs in the 454 paired end library. Illumina sequencing data was assembled with Velvet [30], and the consensus sequences were shredded into 1.5 kb overlapped fake reads and assembled together with the 454 data. The Phred/Phrap/Consed software package (www.phrap.com) was used for sequence assembly and quality assessment in the following finishing process. After the shotgun stage, reads were assembled with parallel phrap (High Performance Software, LLC). Possible mis-assemblies were corrected with gapResolution (http://www.jgi.doe.gov/), Dupfinisher, or sequencing cloned bridging PCR fragments with subcloning or transposon bombing [31]. Gaps between contigs were closed by editing in Consed, by PCR and by Bubble PCR primer walks (J-F. Cheng, unpublished). A total of 297 additional Sanger reactions were necessary to close gaps and to raise the quality of the finished sequence. Illumina reads were also used to improve the final consensus quality using Polisher [32]. The error rate of the completed genome sequence is less than 1 in 100,000.

### Genome annotation

Genes were identified using Prodigal [33] as part of the Oak Ridge National Laboratory genome annotation pipeline, followed by a round of manual curation using the JGI GenePRIMP pipeline [34]. The predicted CDSs were translated and used to search the National Center for Biotechnology Information (NCBI) nonredundant database, UniProt, TIGR-Fam, Pfam, PRIAM, KEGG, COG, and InterPro databases. Additional gene prediction analysis and functional annotation was performed within the Integrated Microbial Genomes - Expert Review (IMG-ER) platform [35].

## Genome properties

**Figure 3:**
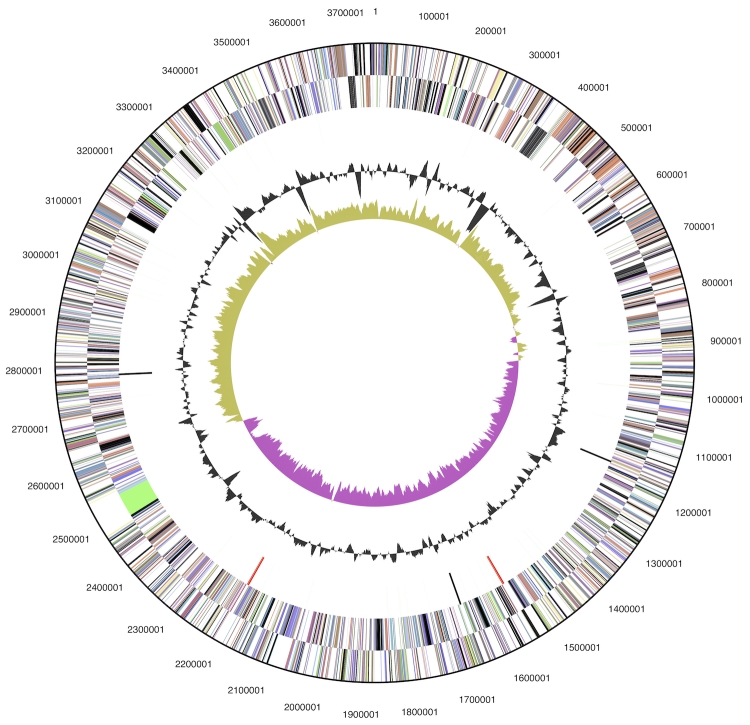
Graphical circular map of the genome. From outside to the center: Genes on forward strand (color by COG categories), Genes on reverse strand (color by COG categories), RNA genes (tRNAs green, rRNAs red, other RNAs black), GC content, GC skew.

The genome is 3,750,771 bp long and comprises one main circular chromosome with a 53.6% GC content (Table 3 and Figure 3). Of the 3,192 genes predicted, 3,137 were protein-coding genes, and 55 RNAs. Seventeen pseudogenes were also identified. The majority of the protein-coding genes (63.6%) were assigned a putative function while the remaining ones were annotated as hypothetical proteins. The distribution of genes into COGs functional categories is presented in Table 4.

**Table 3:** Genome Statistics

**Attribute**	Value	% of Total
Genome size (bp)	3,750,771	100.00%
DNA Coding region (bp)	3,398,430	90.61%
DNA G+C content (bp)	2,010,480	53.60%
Number of replicons	1	
Extrachromosomal elements	0	
Total genes	3,192	100.00%
RNA genes	55	1.72%
rRNA operons	2	
Protein-coding genes	3,137	98.28%
Pseudo genes	17	0.53%
Genes with function prediction	2,031	63.63%
Genes in paralog clusters	355	11.12%
Genes assigned to COGs	2,028	63.53%
Genes assigned Pfam domains	2,174	68.11%
Genes with signal peptides	956	29.95%
Genes with transmembrane helices	755	23.65%
CRISPR repeats	0	

**Table 4:** Number of genes associated with the general COG functional categories

**Code**	**value**	**%age**	**Description**
J	141	6.3	Translation, ribosomal structure and biogenesis
A	0	0.0	RNA processing and modification
K	145	6.5	Transcription
L	109	4.9	Replication, recombination and repair
B	1	0.0	Chromatin structure and dynamics
D	19	0.9	Cell cycle control, mitosis and meiosis
Y	0	0.0	Nuclear structure
V	33	1.5	Defense mechanisms
T	95	4.2	Signal transduction mechanisms
M	163	7.3	Cell wall/membrane biogenesis
N	36	1.6	Cell motility
Z	0	0.0	Cytoskeleton
W	0	0.0	Extracellular structures
U	84	3.7	Intracellular trafficking and secretion
O	93	4.1	Posttranslational modification, protein turnover, chaperones
C	126	5.6	Energy production and conversion
G	156	7.0	Carbohydrate transport and metabolism
E	150	6.7	Amino acid transport and metabolism
F	59	2.6	Nucleotide transport and metabolism
H	116	5.2	Coenzyme transport and metabolism
I	69	3.1	Lipid transport and metabolism
P	163	7.3	Inorganic ion transport and metabolism
Q	46	2.1	Secondary metabolites biosynthesis, transport and catabolism
R	285	12.7	General function prediction only
S	157	7.0	Function unknown
-	1,164	36.5	Not in COGs

## Insights from genome sequence

With 94% identity based on 16S rRNA analysis ‘*F. fucoidanolyticus’* is one of the closest related, cultivated organism to *C. akajimensis*. Sakai and colleagues report the existence of intracellular α-L-fucosidases and sulfatases, which enable ‘*F. fucoidanolyticus’* to degrade fucoidan [14]. This fucoidan degrading ability could be shared by *C. akajimensis*, as the annotation of the genome sequence revealed the existence of 49 sulfatases and 12 α-L-fucosidases belonging to glycoside hydrolase family 29. Furthermore 12 β-agarases are encoded in the genome of *C. akajimensis*, which is not in accordance to Yoon *et al*., who reported that agar was not hydrolyzed by *C. akajimensis* [1]. Forty-two genes coding for transcriptional regulators belonging to the AraC-family were found in *C. akajimensis*. It might be noteworthy that the genes coding for the AraC-family regulators, agarases, sulfatases and α-L-fucosidases are unequally distributed over the genome, with most of them localized in the first third of the genome (bp 33,731-1,412,308). The genes for several fucosidases and sulfatases are clustered and their expression might be under the control of an AraC-family regulator.

In addition to *C. akajimensis* only two more genomes of members of the *Opitutae* are sequenced (but not yet published): *Opitutus terrae,* an obligately anaerobic, motile bacterium isolated from a rice paddy soil microcosms [6] and *Opitutaceae* bacterium TAV2 isolated from the gut of a wood-feeding termite. Because of the quite distant relatedness of these three sequenced organisms, a comparison of genomes seems to be of limited use. The reported characteristic differences between the *Opitutae* [1] are partly reflected in the now known genome sequence. In the case of the motile bacterium *O. terrae* 36 proteins belonging to the COG pathway ‘flagellum structure and biogenesis’ are predicted, whereas in the genome of the non-motile *C. akajimensis,* no proteins belonging in this category are encoded. Another characteristic feature is the ability to reduce nitrate. In both genomes genes encoding for nitrate reductase (EC: 1.7.99.4: *O. terrae* Oter_1740, *C. akajimensis* Caka_0064, Caka_0348) and nitrite reductase are predicted (EC: 1.7.7.1: *O. terrae* Oter_1737, *C. akajimensis* Caka_0346; EC: 1.7.2.2: *O. terrae* Oter_4608, *C. akajimensis* Caka_2912), but only for *O. terrae* nitrate reduction is reported [14]. In the case of starch hydrolysis, the genome data match the experimental data previously reported. The *O. terrae* reported to be starch-hydrolyzing encodes one α-amylase and for three proteins containing α-amylase domains. For *C. akajimensis,* starch hydrolysis is not reported and in the genome there is only one gene identified that could encode for an α-amylase.
